# Impact of Quadriceps Muscle Fatigue on Ankle Joint Compensation Strategies During Single-Leg Vertical Jump Landing

**DOI:** 10.3390/s24206712

**Published:** 2024-10-18

**Authors:** Chen Chen, Huiyu Zhou, Datao Xu, Xiangli Gao, Liangliang Xiang, Yaodong Gu

**Affiliations:** 1 Faculty of Sports Science, Ningbo University, Ningbo 315211, China; chenchen16@aliyun.com (C.C.); zhouhuiyu@nbu.edu.cn (H.Z.); xudatao3@gmail.com (D.X.); gaoxiangli1@aliyun.com (X.G.); 2 Faculty of Engineering, University of Pannonia, 8201 Veszprem, Hungary; 3 KTH MoveAbility Lab, Department of Engineering Mechanics, KTH Royal Institute of Technology, SE-100 44 Stockholm, Sweden; 4 Faculty of Engineering, University of Szeged, 6720 Szeged, Hungary

**Keywords:** fatigue, ankle injuries, landing, compensatory strategies, lower limb joints

## Abstract

This study investigates the impact of quadriceps fatigue on lower limb biomechanics during the landing phase of a single-leg vertical jump (SLJ) in 25 amateur male basketball players from Ningbo University. Fatigue was induced through single-leg knee flexion and extension exercises until task failure. Kinematic and dynamic data were collected pre-fatigue (PRF) and post-fatigue (POF) using the Vicon motion capture system and the AMTI force platform and analyzed using an OpenSim musculoskeletal model. Paired sample *t*-tests revealed significant changes in knee and hip biomechanics under different fatigue conditions, with knee joint angle (*p* < 0.001), velocity (*p* = 0.006), moment (*p* = 0.006), and power (*p* = 0.036) showing significant alterations. Hip joint angle (*p* = 0.002), moment (*p* = 0.033), and power (*p* < 0.001) also exhibited significant changes. Muscle activation and joint power were significantly higher in the POF condition, while joint stiffness was lower. These findings suggest that quadriceps fatigue leads to biomechanical adjustments in the knee and hip joints, which may increase the risk of injury despite aiding in landing stability.

## 1. Introduction

Single-leg vertical jump (SLJ) landings are critical for halting, changing direction, and preparing for subsequent movements, as the non-supporting leg can quickly engage after landing [1,2]. SLJ places high demands on the ankle joint, which absorbs impact forces ranging from 2.0 to 5.0 times body weight [3,4,5]. The ankle, essential for structural stability and biomechanical support, enables movements like flexion, extension, and rotation [6,7]. Different landing patterns subject lower limb joints and ligaments to varying loading modes, which can impact joint health and performance [8,9,10]. Ankle sprains are among the most common injuries, accounting for 15% of all sports-related injuries [2,11,12,13,14,15,16,17,18]. High-intensity maneuvers, combined with physical contact in sports, challenge the stability of the ankle joint, increasing the likelihood of injury [19,20,21,22,23].

Joint stiffness ($k_{joint}$) represents the mechanical resistance to displacement within lower limb joints and is influenced by physiological structure, movement type, and mechanical properties [24]. It plays a key role in supporting loads and stabilizing the body during dynamic tasks. Abnormal joint stiffness can increase the risk of injury—insufficient stiffness may result in excessive joint mobility, while excessive stiffness can raise the risk of bone injuries [25,26,27,28]. Previous studies have highlighted the importance of exploring the relationship between joint stiffness and leg stiffness across various motor tasks and speeds, especially in high-impact activities like jumping and running [29]. Investigating joint stiffness during landing mechanics under fatigue may provide deeper insights into injury mechanisms and prevention strategies.

Fatigue has been shown to negatively affect motor control, joint position sense, and muscle response, leading to improper positioning and a decreased ability to respond quickly to movement changes, predisposing individuals to injuries [30]. Evidence indicates that fatigue can cause malalignment and compromise knee joint stability during athletic activities [31,32,33]. Under fatigue conditions, athletes experience increased impact acceleration, characterized by rapid deceleration of the tibia during landings [34]. Fatigue affects lower-extremity angles and ground reaction forces, altering landing strategies and impairing impact attenuation [35,36]. Thus, assessing the impact of fatigue on landing mechanics is essential for developing effective injury prevention strategies.

The quadriceps muscles are crucial for lower limb strength and function, and their maximal force-generating capacity is vital in high-intensity activities such as sprinting and maximal cycling [37]. Individuals with greater quadriceps force can achieve higher speeds and produce more power [38,39,40]. However, research on localized fatigue’s effects on lower limb biomechanics remains limited. Previous studies have examined the impact of fatigue in other muscle groups on SLJ landing mechanics, demonstrating that such fatigue affects angular velocities and increases injury risk [41]. For instance, gluteus maximus fatigue alters muscle coordination during single-leg landings and increases hamstring load, potentially leading to strains [42].

The main objective of this study is to systematically investigate biomechanical changes in the ankle joint under conditions of quadriceps muscle fatigue. While previous studies have explored quadriceps fatigue in cycling and static tasks, its impact on the dynamic landing phase of SLJ remains underexplored. Given the quadriceps’ crucial role in stabilizing the knee joint and overall lower limb strength [37], it is essential to understand how quadriceps fatigue influences SLJ landing mechanics, particularly concerning changes in ankle joint strategies. The aim was to investigate the impact of quadriceps muscle fatigue on lower limb biomechanics, with a specific focus on compensation strategies during the SLJ landing phase. We hypothesize that compared to the PRF condition, the POF phase will show differences in the angles, velocities, moments, and power of the ankle, knee, and hip joints, as well as in muscle activation.

When fatigue sets in, the central nervous system initiates compensatory mechanisms to maintain performance and prevent injury. These compensations, particularly in the ankle joint, are crucial for maintaining balance during dynamic movements like SLJ [43]. However, over time, altered joint mechanics, such as reduced ankle stability and increased reliance on the hip, may predispose individuals to chronic overuse injuries [44]. Understanding these compensatory mechanisms can inform injury prevention strategies and optimize training and rehabilitation programs to improve performance while minimizing injury risk.

## 2. Materials and Methods

### 2.1. Participants

Based on prior research, we determined the required sample size using G-Power software (version 3.1.9.7; Heinrich Heine University, Düsseldorf, Germany). A paired sample *t*-test was conducted, with an effect size of 0.6, a power of 0.8, and a significance level of 0.05, to perform the analysis of variance (ANOVA) [45]. Thus, the study recruited 25 amateur male basketball athletes (age 23.42 ± 1.14 years, height 176.11 ± 4.95 cm, body weight 71.78 ± 7.17 kg) from Ningbo University. To ensure participant standardization, specific inclusion criteria were applied: (1) all participants were young, healthy amateur basketball players from Ningbo University; (2) each participant played basketball at least three times per week, with each session lasting a minimum of two hours; (3) none of the participants had sustained lower limb injuries in the past six months, nor had any medical conditions that could potentially affect the experimental results; (4) no participants had undergone prior surgery on their lower limbs. Before data collection, participants were thoroughly informed about the study’s objectives, procedures, conditions, and requirements. Detailed information was provided in a written format. The study was approved by the Ethics Committee of Ningbo University, and informed consent was obtained from all subjects.

### 2.2. Experimental Procedure

The experiment was conducted in the Sports Biomechanics Laboratory at Ningbo University. Building on prior research, we meticulously affixed 38 standard markers, each with a diameter of 12.5 mm, to the participants to accurately capture their motion trajectories [46]. A Vicon system with 8 cameras (Oxford Metric Ltd., Oxford, UK) was employed to collect kinematic data, sampled at a frequency of 200 Hz. During the SLJ landing task, the force platform (AMTI, Watertown, MA, USA) was set to a sampling frequency of 1000 Hz to collect kinetic data [47]. Both experimental setups were synchronized. Surface electromyography (EMG) sensors (Delsys, Boston, MA, USA) were attached to the muscle bellies of the subjects’ vastus lateralis (VL), vastus medialis (VM), rectus femoris (RF), biceps femoris (BF), lateral gastrocnemius (LG), Soleus (SOL), medial gastrocnemius (MG), and tibialis anterior (TA) to measure muscle activation [46]. The placement of EMG sensors was conducted in accordance with the guidelines established by the Surface EMG for Noninvasive Assessment of Muscles (SENIAM) [48]. Additionally, Maximum Voluntary Contractions (MVCs) were recorded for each of the eight muscle groups to standardize muscle activation measurements.

Pre-Fatigue Landings. Before the formal experiment, participants began by warming up on a treadmill for 10 min at a speed of 8 km/h. Following this, they performed stretching exercises to ensure they could perform at their maximum potential during the experiment. All participants wore tight-fitting shorts and shirts. Subsequently, they familiarized themselves with the experimental environment and protocols, and they practiced single-leg vertical jumping before the formal testing began. To minimize errors caused by variations in participants’ flight time and distance, all participants were instructed to take off and land on the force plate. During the formal experiment, participants provided a set of static data [1]. They were directed to stand in an anatomical position, place one foot on the force plate, and prepare for data collection upon hearing a command. Data collection commenced when the ground reaction force exceeded 10 N [49]. Participants performed 10 non-fatigued single-leg jumps landing on the force platform. Throughout the experiment, investigators closely monitored participant performance. If a participant failed to fully place their foot on the force plate, the trial was deemed invalid, necessitating repeated measurements to ensure data accuracy and reliability.

**Figure 1:**
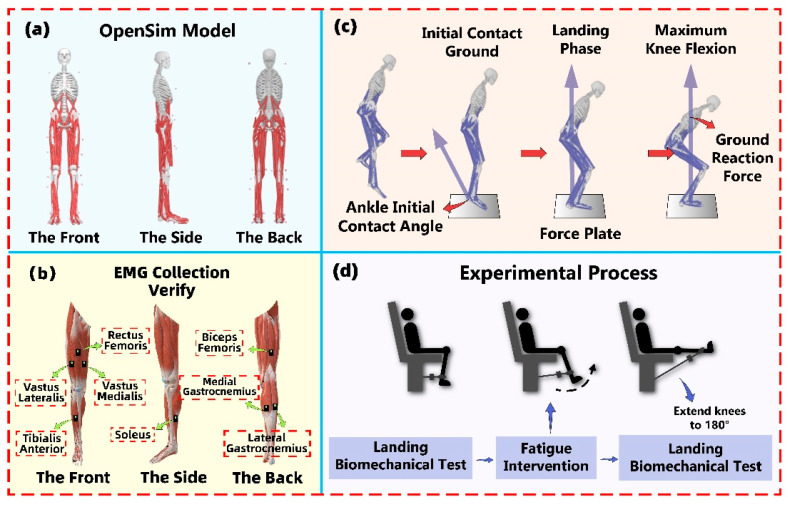
An overview of the musculoskeletal model and overall experimental procedure of the study. (**a**) An illustration of the position of the reflective marking points in the constructed musculoskeletal model. (**b**) An illustration of the placement of EMG sensors on human lower limbs. (**c**) An illustration of the SLJ test process, detailing the landing phase from initial ground contact to maximum knee flexion. (**d**) An illustration of the entire experimental process, consisting of three main steps: (1) SLJ biomechanics test before fatigue intervention; (2) fatigue intervention; (3) SLJ biomechanics test after fatigue intervention.

Post-Fatigue Landings. Following the 10 non-fatigued landings, participants underwent a fatigue protocol specific to the landing test session. To ensure that the post-fatigue assessments accurately reflected fatigue, participants immediately performed the post-fatigue landing protocol within 30 s of completing the fatiguing exercises, with no rest permitted between the post-fatigue landing trials. A functional open kinetic chain exercise protocol was employed to induce lower limb muscle fatigue. Participants performed repetitive knee flexion and extension exercises using resistance bands, with resistance tailored to their individual strength. Each movement was executed within a knee joint flexion range of 90° to 180°. Participants started with the knee flexed at 90° to form a right angle and then extended the knee to approximately 180° (Figure 1d). To ensure trunk stability, participants were provided with clear posture instructions before the experiment, requiring them to sit against the backrest of the chair and grip the side handles firmly to limit unnecessary trunk movements. Movement frequency was regulated by having participants perform the repetitive flexion and extension exercises in time with a digital metronome set at 60 beats per minute. Participants were required to maintain a consistent movement rate during both the concentric (flexion) and eccentric (extension) phases of the exercise. The fatigue protocol was adapted from Yates et al. [50], which investigated elbow flexor fatigue. The protocol was modified for knee flexion and extension tasks by adjusting the movement frequency and the knee joint range of motion. Each participant performed the flexion and extension exercises until the onset of fatigue, defined as the point when they either fell behind the set pace by four flexion–extension cycles or failed to complete two consecutive cycles. Before starting the protocol, participants were instructed to prioritize fully extending the knee and lightly contacting the range of motion block during knee flexion, even if it meant falling behind the prescribed rhythm [51].

### 2.3. Data Processing and Analysis

This study investigated the alterations in lower limb biomechanical parameters during the landing phase of the single-leg vertical jump in response to a fatigue intervention. Vicon Nexus software (v1.8.5, Vicon Metrics Ltd., Oxford, UK) exports data in C3D format to acquire participants’ kinematic and kinetic data [46]. Then, we imported all of the data into MATLAB ( R2022a, The MathWorks, Natick, MA, USA) and processed them through a written script, involving operations such as coordinate transformation, low-pass filtering, data extraction, and format conversion. The vertical ground reaction force (VGRF) value of the force platform exceeds 10 N, which we define as the initial point [47]. Data acquisition begins at the initial point of contact and ends with maximum knee flexion. The choice of filter frequency is based on previous research by Winter [52]. Dynamic residual analysis is performed in a subset to confirm the most appropriate SNR (signal-to-noise ratio). The VGRF and kinematics data were filtered using fourth-order zero-phase lag Butterworth low-pass filters with cutoff frequencies of 20 Hz and 10 Hz [19]. Kinematic and ground reaction force data were extracted and converted into trc format (marker trajectories) and force plate data format, both of which are essential for musculoskeletal simulations.

In this study, OpenSim (v4.4, Stanford University, Stanford, CA, USA) was used for the processing and calculation of biomechanical parameters. We imported the static model into OpenSim and utilized the scale tool to acquire a body measurement model for each participant. The muscle origin and insertion point were identified to align with each participant’s limb length. Using the Inverse Kinematics (IK) tool in OpenSim 4.4 software, we calculated the joint angle and created a motion file (mot) for the landing phase of a single-leg vertical jump. The residual reduction algorithm (RRA) was used to smooth the kinematic data, which improved the accuracy of the dynamic data and made them consistent with the measured data. Muscle activation is determined through static optimization. The EMG activation variables are qualitatively compared with the muscle activation simulated by OpenSim to evaluate the reliability of the OpenSim model. Joint powers were calculated as the product of joint moments and velocities. During the landing phase, positive, negative, and absolute (sum of positive and negative) work at the hip, knee, and ankle joints was assessed by numerically integrating power over time. Impulse (force–time integral) represents the cumulative effect of plantar force during the landing phase of a single-leg vertical jump. Joint stiffness was calculated by dividing the change in joint moment (ΔM) by the change in joint angle (Δθ) during the landing phase. Building on prior research, we utilized the following formula [53,54,55]:

(1) $$k_{joint}=\Delta M/\Delta \theta ,$$

where ΔM represents the change in joint moment, and Δθ represents the change in joint angle.

### 2.4. Statistical Analysis

The landing phase is defined as maximum knee flexion after initial contact with the surface. All data were subjected to a Shapiro–Wilk normality test to evaluate whether the data conform to a normal distribution prior to statistical analysis. Subsequently, a paired sample *t*-test was used to carefully examine the adaptive changes in ankle strategy caused by quadriceps fatigue. In the statistical parametric mapping (SPM) analysis, all kinematic and kinetic data from the landing phase were extracted, and a custom MATLAB script was employed to interpolate the data points into a time-series curve with 101 data points, representing the full 0% to 100% span of the landing phase. After preparing the data, statistical analysis was conducted using the open-source SPM 1d paired samples *t*-test script, with a significance threshold set at *p* < 0.05 [56,57].

## 3. Results

### 3.1. Joint Kinematics

**Figure 2:**
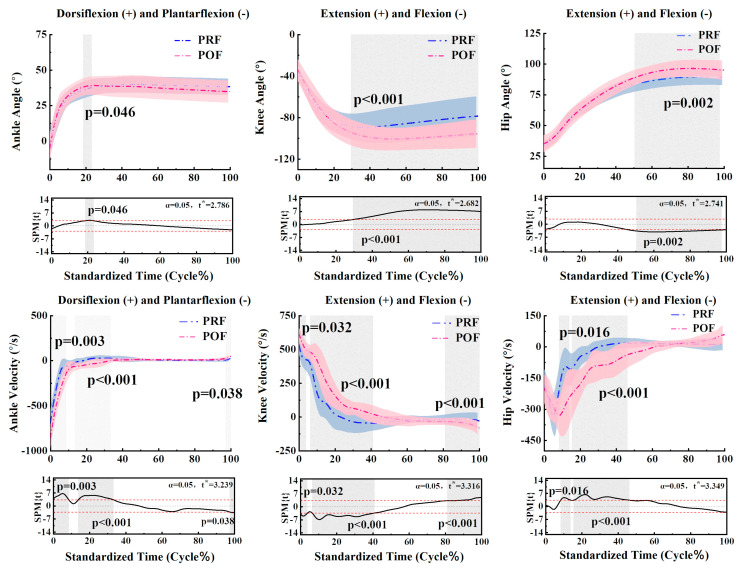
An illustration of the results between PRF and POF of the lower limb showing the statistical parametric mapping outputs for the joint kinematics during the landing phase. The blue and pink lines and areas denote the mean and standard deviation in the pre-fatigue and post-fatigue conditions, respectively. The t* values are shown on the right side of each image. Gray shades indicate significant differences and the t-values of the SPM for all participants, while dashed red lines represent the results at *p* = 0.05.

The SPM analysis revealed differences in joint kinematics during the landing phase when comparing PRE and POF conditions. Figure 2 displays the significant differences in ankle, knee, and hip angles and velocities during the landing phase between PRF and POF conditions. For the ankle, significant differences were observed in angle (18.64–23.31%, *p* = 0.046) and velocity (0–8.5%, *p* = 0.003) (13.7–33.2%, *p* < 0.001) (97.3–100%, *p* = 0.038). For the knee, significant differences were found in angle (29.49–100%, *p* < 0.001) and velocity (0–3.82%, *p* = 0.032) (6.41–41%, *p* < 0.001) (81.1–100%, *p* < 0.001). For the hip, significant differences were noted in angle (50.48–97.48%, *p* = 0.002) and velocity (8.28–13.79%, *p* = 0.016) (15.43–46.15%, *p* < 0.001). Table 1 indicates that during the SLJ landing phase, there are significant differences between the PRF and POF phases in ankle plantarflexion velocity (*p* < 0.001). Additionally, significant differences were observed in the knee flexion angle (*p* < 0.001) and knee extension velocity (*p* = 0.006). Finally, significant differences were found in hip extension angle (*p* = 0.002).

### 3.2. Joint Kinetics

**Figure 3:**
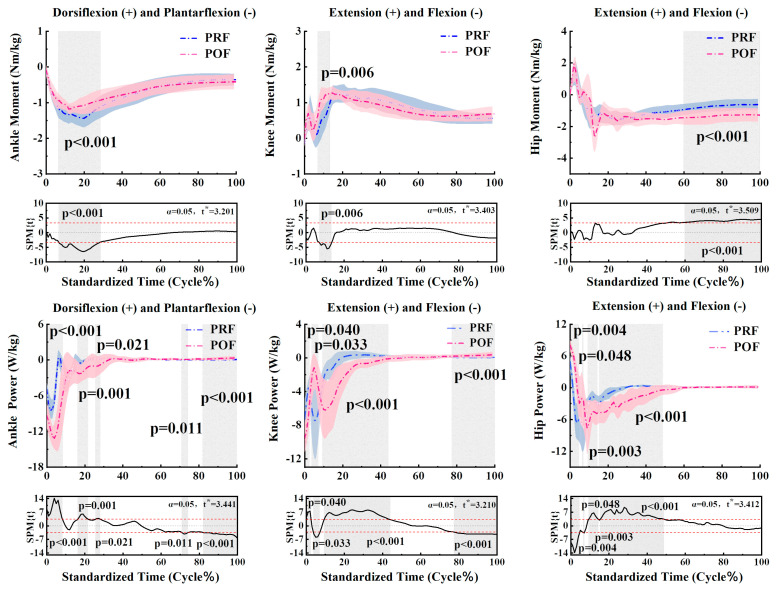
The descriptive results between PRF and POF lower limb statistical parametric mapping for the change in joint kinetics during the landing phase. The blue and pink lines and areas denote the mean and standard deviation in the pre-fatigue and post-fatigue conditions, respectively. The t* values are shown on the right side of each image. Gray shades highlight significant differences and the t-values from the SPM analysis for all participants, whereas the dashed red lines represent *p* = 0.05.

The SPM analysis highlighted significant differences in the kinetic parameters of the ankle, knee, and hip joints during the landing phase. Figure 3 illustrates these notable variations in the moment and power of the ankle, knee, and hip joints between PRF and POF conditions. For the ankle, significant differences were observed in moment (6.5–28.6%, *p* < 0.001) and power (0–8.27%, *p* < 0.001) (16.27–21.71%, *p* = 0.001) (25.6–28.1%, *p* = 0.021) (70.85–74.17%, *p* = 0.011) (82.02–100%, *p* < 0.001). For the knee, significant differences were found in moment (7.01–13.44%, *p* = 0.006) and power (0–2.54%, *p* = 0.040) (3.74–7.18%, *p* = 0.033) (9.36–44.05%, *p* < 0.001) (77.44–100%, *p* < 0.001). For the hip, significant differences were noted in moment (59.87–100%, *p* < 0.001) and power (0–4.64%, *p* = 0.004) (6.54–7.08%, *p* = 0.048) (9.38–14.41%, *p* = 0.003) (15.41–48.82%, *p* < 0.001). Table 1 reveals the significant differences in ankle plantarflexion moment (*p* = 0.002), ankle dorsiflexion power (*p* = 0.014), and ankle plantarflexion power (*p* < 0.001). Additionally, significant differences were observed in knee flexion moment (*p* = 0.006) and knee extension power (*p* = 0.036). Finally, notable differences were noted in hip extension power (*p* < 0.001).

### 3.3. Muscle Activation

**Figure 4:**
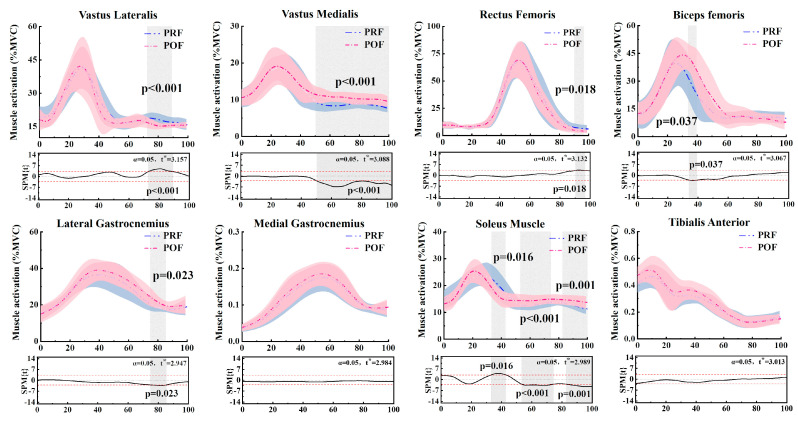
An illustration of the results comparing PRF and POF conditions of the lower limb, showing the statistical parametric mapping (SPM) outputs for the vastus lateralis, vastus medialis, rectus femoris, biceps femoris, lateral gastrocnemius, soleus, medial gastrocnemius, and tibialis anterior angles during the landing phase. The blue and pink lines and areas denote the mean and standard deviation in the pre-fatigue and post-fatigue conditions, respectively. The t* values are displayed to the left of each image. Gray shading highlights areas of significant differences and t-values across all participants, while dashed red lines indicate the threshold at *p* = 0.05.

The SPM analysis indicated differences in muscle activation during the landing phase when comparing PRF and POF conditions. Significant activation differences were observed in the vastus lateralis (72.72–89.15%, *p* < 0.001), vastus medialis (50.23–100%, *p* < 0.001), rectus femoris (90.23–96.79%, *p* = 0.018), biceps femoris (34.05–39.51%, *p* = 0.037), lateral gastrocnemius (74.74–85.44%, *p* = 0.023), and Soleus muscle (33.08–42.87%, *p* = 0.016; 53.31–74.49%, *p* < 0.001; 82.64–100%, *p* = 0.001). In contrast, no significant activation differences were found in the medial gastrocnemius and tibialis anterior between PRF and POF conditions during the landing phase. Figure 4 highlights the differences in the activation of the Vastus lateralis, vastus medialis, rectus femoris, biceps femoris, lateral gastrocnemius, Soleus, medial gastrocnemius, and tibialis anterior muscles between PRF and POF conditions during the landing phase. Table 2 highlights significant differences in the biceps femoris (*p* = 0.030) and tibialis anterior (*p* < 0.001) between PRF and POF conditions.

### 3.4. Impact Load

**Figure 5:**
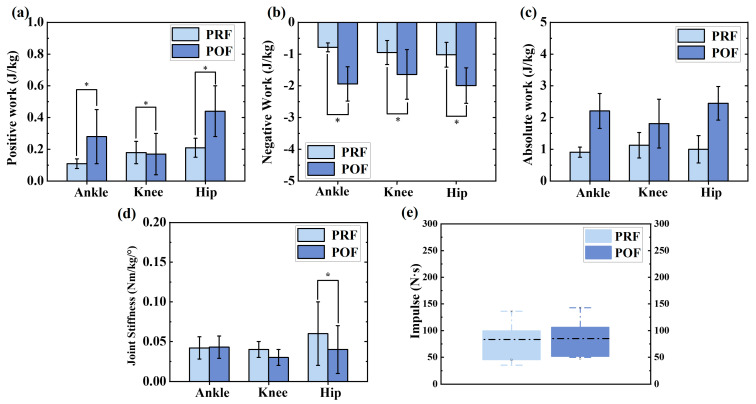
The changes in joint work, joint stiffness, and impulse between PRF and POF of the lower limb during the SLJ landing phase. (**a**) Positive work; (**b**) negative work; (**c**) absolute work; (**d**) joint stiffness calculated for the SJL landing phase. (**e**) Impulse (force-time integral) is calculated as the area under the GRF curve measured during the landing phase. “*” indicates a significant difference between PRF and POF conditions (*p* < 0.05).

Figure 5 shows the difference in joint work, joint stiffness, and impulse between PRF and POF phases during the SLJ landing phase, which collectively influences the impact load experienced by the lower limb joints. Significant differences were observed in the sagittal plane for positive, negative, and absolute work during landing. Specifically, for positive work, significant differences were noted at the hip (*p* < 0.001) and ankle (*p* < 0.001). For negative and absolute work, significant differences were found at the hip (*p* < 0.001), knee (*p* < 0.001), and ankle (*p* < 0.001). These variations in joint work suggest differential energy absorption and transfer, contributing to variations in the impact load across these joints. Additionally, while significant differences in hip and knee joint stiffness were observed between PRF and POF conditions, indicating altered mechanical responses that could influence impact load distribution, the overall landing impulse did not show a significant difference. This suggests that despite changes in joint stiffness and work, the total force exerted over time during landing remained relatively consistent, potentially modulating the effective impact load experienced by the joints.

## 4. Discussion

This study aimed to investigate the effects of fatigue on lower limb biomechanics during the landing phase of the SLJ, focusing on the differences between PRF and POF conditions. We initially hypothesized that fatigue could influence ankle joint dynamics, prompting adjustments in the strategies of the knee and hip joints during landing. Our findings confirmed this hypothesis and revealed a more pronounced plantarflexion angular velocity, moment, and power in the ankle joint during the POF phase compared to PRF. Moreover, the significant differences in joint work observed between PRF and POF, along with the notable reduction in hip and knee joint stiffness in the POF phase compared to PRF, corroborate our initial assumptions. These outcomes provide a comprehensive understanding, from a biomechanical perspective, of the compensatory strategies the body employs to maintain stability and mitigate the risk of injury during dynamic movements [19].

Previous research indicates that fatigue reduces ankle joint stability and alters moment and power output during landing [58]. Our findings are consistent with this, demonstrating that POF significantly decreases ankle joint angle and velocity while increasing moment and power compared to PRF. This suggests that fatigue impairs the ankle’s ability to control movement and generate force, which affects force management and transmission. Additionally, previous studies have indicated that reduced force generation in the fatigued ankle may contribute to increased biomechanical stress, which could potentially elevate the risk of injury [59]. Future research should include frontal plane analysis to provide a more comprehensive understanding of these dynamics. 

Furthermore, our results show that fatigue increases knee joint flexion angle and moment, likely due to changes in movement strategy [60]. We also observed increased knee flexion angle and moment, alongside decreased extension velocity and increased extension power in the POF phase compared to PRF. We hypothesize that these changes result from reduced muscle strength and compensatory strategies to maintain balance. The observed increase in hip extension angle may serve as a compensatory mechanism to stabilize the body during landing. These findings are in line with reports that fatigue leads to significant changes in hip joint kinematics and power output [61]. Overall, these fatigue-induced changes in joint parameters may contribute to an increased risk of lower limb injuries [41].

Significant differences in muscle activation were noted between PRF and POF conditions during the SLJ landing phase. Specifically, muscles such as the vastus lateralis, vastus medialis, rectus femoris, biceps femoris, lateral gastrocnemius, and Soleus showed increased activation in the POF condition. This heightened muscle activation is crucial for maintaining stability and managing impact forces during landing, representing a compensatory response to the impairments caused by fatigue. While these compensatory mechanisms may help sustain stability, they can also elevate injury risk due to altered force distribution and increased muscle load. Additionally, the differences in activation levels and patterns among other lower extremity muscles contribute to vertical support during landing [61]. Participants are reported to possess an increased quadriceps moment with increasing knee flexion during single-leg landings [62,63]. While these compensatory mechanisms help sustain stability, they may also elevate injury risk due to altered force distribution and increased muscle load [64]. Overall, the changes in muscle activation due to fatigue underscore the importance of targeted interventions to improve muscle endurance and control in high-intensity activities.

We observed significant differences in joint work, stiffness, and impulse between the PRF and POF phases, collectively influencing the impact load on lower limb joints. During the landing phase, joint work was calculated by integrating power over time, showing an increase in negative joint work during the POF phase, with eccentric movements dominating this phase [61]. This increase in absolute total work suggests higher energy demands in the fatigued state, particularly at the hip and ankle joints, which exhibited notable variations in positive work. This increase suggests higher energy demands in the fatigued state, particularly at the hip and ankle joints, indicating a compensatory mechanism aimed at absorbing increased energy due to quadriceps fatigue [65]. 

Moreover, changes in hip and knee joint stiffness highlight the lower limb’s adaptive mechanical responses to fatigue. Increased stiffness in these joints during the POF phase may serve as a protective strategy by redistributing the impact load away from the ankle joint. However, the observed reduction in stiffness suggests a decreased ability to resist joint displacement, potentially raising the risk of knee injuries [66]. Despite these changes in joint stiffness and work, the overall landing impulse remained consistent between the phases, indicating effective regulation of muscle activation even under fatigue. This consistent impulse plays a crucial role in maintaining the effective impact load on the joints within safe limits, thus preventing excessive strain on the ankle joint during landing.

These results imply that quadriceps fatigue induces a biomechanical adjustment that influences the entire lower limb kinetic chain. The ankle joint, although affected by fatigue-induced changes in the proximal joints, may rely on consistent impulse and altered joint stiffness to compensate for the reduced efficiency of the fatigued quadriceps, as muscle and joint stiffness are critical for stability during high-intensity tasks [67]. This compensation strategy underscores the importance of considering the interconnectedness of lower limb joints when assessing the impact of muscle fatigue on injury risk and performance during dynamic movements such as SLJ landing.

However, several limitations were identified in this study. Firstly, the current study focused solely on sagittal plane kinematics and kinetics. A multi-planar dynamics analysis may aid in future injury prevention studies. Furthermore, biomechanical data for landing were only collected under pre-fatigue and post-fatigue conditions. Future research should consider evaluating various fatigue levels (mild, moderate, and severe) to better understand the trends and thresholds of significant changes. Secondly, the sample consisted exclusively of male basketball players. To reduce sampling bias, future studies could explore the impact of fatigue on female basketball players or athletes at different performance levels. Lastly, fatigue may impair participants’ ability to control their trunk, and the resultant shifts in the center of mass due to trunk movement could affect the outcomes.

## 5. Conclusions

This study examined the effects of fatigue on lower limb biomechanics during the landing phase of the SLJ, specifically comparing pre-fatigue (PRF) and post-fatigue (POF) conditions. While we initially proposed that quadriceps fatigue might be associated with an increased risk of ankle sprains, our findings emphasize that quadriceps fatigue leads to significant biomechanical changes in ankle joint mechanics and overall landing strategies. The observed increase in dorsiflexion angle, along with alterations in joint velocities and moments, suggests adaptive responses aimed at mitigating potential injury risks. However, the direct relationship between these biomechanical changes and ankle sprain risk requires further exploration. Future research should incorporate a comprehensive three-dimensional motion analysis and investigate lower extremity biomechanics under various fatigue levels and exercise conditions. Additionally, increasing the sample size will be essential for validating our findings and understanding the broader implications of fatigue on injury risk.

## Figures and Tables

**Table 1:** Comparison of changes in all joint angle, velocity, moment, and power variables between PRF and POF conditions during SLJ landing phase.

	Parameters	PEAK VALUE	PRF Mean ± SD	POF Mean ± SD	*p*-Value
Ankle	Angle (°)	Dorsiflexion	41.16 ± 6.11	41.43 ± 5.34	0.765
		Plantarflexion	−5.11 ± 8.80	−3.50 ± 9.73	0.174
	Velocity (°/s)	Dorsiflexion	53.18 ± 34.05	51.89 ± 16.60	0.877
		Plantarflexion	−698.07 ± 150.38	−852.3 ± 137.89	<0.001 *
	Moment (Nm/kg)	Dorsiflexion	−0.10 ± 0.09	−0.08 ± 0.13	0.698
		Plantarflexion	−1.65 ± 0.25	−1.38 ± 0.15	0.002 *
	Power (W/kg)	Dorsiflexion	0.91 ± 0.46	1.81 ± 1.52	0.014 *
		Plantarflexion	−8.87 ± 1.62	−14.18 ± 1.17	<0.001 *
Knee	Angle (°)	Extension	−36.22 ± 6.20	−34.31 ± 10.70	0.071
		Flexion	−94.50 ± 10.28	−104.03 ± 11.06	<0.001 *
	Velocity (°/s)	Extension	543.95 ± 56.82	608.47 ± 44.67	0.006 *
		Flexion	−89.57 ± 58.82	−92.89 ± 39.38	0.882
	Moment (Nm/kg)	Extension	1.49 ± 0.23	1.47 ± 0.14	0.792
		Flexion	−0.29 ± 0.13	−0.11 ± 0.18	0.006 *
	Power (W/kg)	Extension	0.70 ± 0.30	0.99 ± 0.65	0.036 *
		Flexion	−9.78 ± 4.13	−9.88 ± 1.99	0.903
Hip	Angle (°)	Extension	89.87 ± 6.63	97.96 ± 7.16	0.002 *
		Flexion	33.40 ± 3.74	35.13 ± 7.11	0.412
	Velocity (°/s)	Extension	41.46 ± 8.69	67.43 ± 39.25	0.123
		Flexion	−387.32 ± 42.29	−408.04 ± 60.31	0.260
	Moment (Nm/kg)	Extension	1.62 ± 0.72	2.17 ± 0.66	0.033 *
		Flexion	−2.42 ± 0.55	−3.54 ± 0.88	0.003
	Power (W/kg)	Extension	4.89 ± 1.57	8.08 ± 1.10	<0.001 *
		Flexion	−11.62 ± 2.73	−10.76 ± 2.67	0.267

Note: “*” indicates a significant difference between PRF and POF conditions in the SLJ landing phase (*p* < 0.05).

**Table 2:** Comparison of changes in all muscle activation variables between PRF and POF conditions during SLJ landing phase.

Peak Muscle Activation	PRF	POF	*p*-Value
(%MVC)	Mean ± SD	Mean ± SD	
Vastus Lateralis	43.33 ± 10.11	46.40 ± 10.89	0.279
Vastus Medialis	20.62 ± 3.03	21.31 ± 5.21	0.573
Rectus Femoris	72.56 ± 16.41	71.08 ± 16.44	0.689
Biceps Femoris	45.49 ±12.34	49.49 ± 9.90	0.030 *
Lateral Gastrocnemius	38.48 ± 7.60	41.73 ± 5.65	0.126
Medial Gastrocnemius	19.37 ± 3.68	20.45 ± 2.76	0.205
Soleus Muscle	25.67 ± 3.91	26.71 ± 4.13	0.340
Tibialis Anterior	6.63 ± 2.36	7.50 ± 3.52	<0.001 *

Note: “*” indicates a significant difference between PRF and POF conditions during the SLJ landing phase (*p* < 0.05); %MVC: percentage of Maximum Voluntary Contraction.

## Data Availability

The data supporting this study’s findings can be obtained from the corresponding author upon reasonable request. However, the data are not publicly accessible due to privacy or ethical considerations.
